# Knowledge, attitudes, and practices of coronary heart disease patients towards cardiac rehabilitation: A cross-sectional study

**DOI:** 10.1097/MD.0000000000044375

**Published:** 2025-09-05

**Authors:** Weifeng Zhang, Haiyan Jia, Yanling Li, Zesheng Xu, Jianjun Chen, Yeran Zhu, Xinwei Jia, Yanmin Wu, Jing Zhang, Yugang Zu

**Affiliations:** a Tianjin Medical University, Tianjin, China; b Department of Cardiology, Affiliated Hospital of Hebei University, Baoding, China; c Department of Cardiovascular Medicine, Cangzhou Central Hospital, Cangzhou Teaching Hospital of Tianjin Medical University, Tianjin, China; d Cardiovascular Department of Baoding First Central Hospital, Baoding, China.

**Keywords:** attitude, cardiac rehabilitation, coronary heart disease, knowledge, patient, practice

## Abstract

This study investigated knowledge, attitudes, and practices of coronary heart disease patients towards cardiac rehabilitation. This cross-sectional study was conducted at Department of Cardiology, Affiliated Hospital of Hebei University from May to June 2024. Demographic data and knowledge, attitudes, and practices scores were evaluated through questionnaires. Of 490 participants enrolled, 292 (59.59%) were male. The mean knowledge, attitude, and practice scores were 11.68 ± 5.21 (range: 0–20), 21.11 ± 2.62 (range: 5–25), and 39.97 ± 7.46 (range: 10–50). Multivariate logistic regression showed knowledge score (OR = 1.08, 95% CI: [1.04–1.13], *P* < .001), attitude score (OR = 1.17, 95% CI: [1.08–1.27], *P* < .001), married status (OR = 5.24, 95% CI: [1.76–15.6], *P* = .003), and myocardial infarction (OR = 1.86, 95% CI: [1.09–3.19], *P* = .023) were independently associated with proactive practice. Structural equation model analysis showed direct effects of knowledge on attitude (β = 0.16, *P* < .001) and practice (β = 0.32, *P* < .001), with attitude impacting practice (β = 0.95, *P* < .001), and knowledge indirectly influencing practice through attitude (β = 0.15, *P* < .001). Coronary heart disease patients showed inadequate knowledge but positive attitudes and practices towards cardiac rehabilitation, highlighting the need for targeted interventions to improve knowledge and engagement in rehabilitation programs.

## 1. Background

Coronary heart disease (CHD) stands as a significant public health concern, with coronary artery disease, also referred to as coronary atherosclerotic heart disease or ischemic heart disease, emerging as the predominant cause of heart attacks and the leading contributor to global mortality among noncommunicable diseases.^[1,2]^ The global burden of CHD is profound, with an estimated 9.4 million fatalities attributed to the condition in 2016 alone, and projections indicate a continued increase in prevalence, expected to rise from 6.8% in 2015 to 8.2% by 2035.^[3]^

In response to the significant health impacts of CHD, cardiac rehabilitation has emerged as a comprehensive secondary prevention intervention.^[4]^ This multifaceted approach encompasses health education, supervised exercise, and psychosocial support, aiming to facilitate recovery, adjustment, and a reduction in the risk of further cardiac complications while improving long-term prognosis.^[5,6]^ Research indicates that participation in cardiac rehabilitation yields positive outcomes, including reduced morbidity and mortality rates.^[7]^ However, despite the well-documented benefits of cardiac rehabilitation, engagement rates remain suboptimal, with only approximately 50% of eligible patients participating on average.^[8]^

Currently, knowledge, attitude, and practices (KAP) surveys serve as diagnostic research tools, shedding light on a group’s understanding, beliefs, and behaviors concerning a specific subject, particularly within the domain of health literacy. These surveys operate under the premise that knowledge positively influences attitudes, which in turn shape behaviors.^[9,10]^ Given the established benefits of cardiac rehabilitation in improving outcomes and reducing the risk of further cardiac events, understanding the perspectives of CHD patients towards these programs is imperative for optimizing their engagement and effectiveness. Despite the recognized advantages of cardiac rehabilitation, participation rates among eligible patients remain suboptimal, underscoring the necessity to identify barriers and facilitators to engagement within this population. Therefore, this study aims to fill this gap by investigating the KAP of CHD patients towards cardiac rehabilitation. Unlike previous research primarily focused on healthcare professionals,^[11,12]^ there is a notable lack of KAP studies targeting Chinese patients. Hence, this study endeavors to address this gap by examining the unique perspectives of CHD patients towards cardiac rehabilitation.

## 2. Methods

### 2.1. Study design and participants

This cross-sectional study was conducted from May 30, 2024 to June 30, 2024 at Affiliated Hospital of Hebei University, targeting patients diagnosed with CHD. The study protocol received ethical approval from the Ethics committee of Affiliated Hospital of Hebei University, and informed consent was obtained from all participants.

Inclusion criteria was patients with CHD, angina pectoris, and acute myocardial infarction, as indicated by coronary angiography showing coronary artery stenosis >50%. Exclusion criteria was patients who refused to participate in the study. A total of 497 patients were initially screened for eligibility. Of these, 5 declined to participate and 2 were excluded due to incomplete questionnaire data. Ultimately, 490 patients were included in the final analysis.

Questionnaires were administered to the study participants through a specialized online survey platform called “Questionnaire Star” (Changsha Ranxing Information Technology Co., Ltd., Changsha, Hunan Province, China). Participants completed the online questionnaires by scanning the QR code provided in WeChat APP group, QQ APP group, department display board and consulting room.

### 2.2. Sample size calculation

The sample size was calculated using the standard formula:


$$\begin{array}{l} n={\left(\frac{Z_{1-\alpha /2}}{\delta }\right)}^{2}\times p\times \left(1-p\right) \end{array}$$

With a confidence level of 95% (Z_1−α/2_ = 1.96) and an expected proportion of 50% (*P* = .5), yielding a minimum required sample size of 384. To account for an anticipated effective response rate of 80%, the final target was set to collect at least 480 questionnaires.

### 2.3. Questionnaire introduction

The questionnaire design was informed by relevant literature.^[13,14]^ Subsequent to the initial design phase, adjustments were made based on feedback from field experts, and a pilot study was conducted involving a small sample size of 42 respondents. The overall reliability of the questionnaire was assessed using Cronbach α coefficient, yielding a value of 0.874.

Comprised of 4 main sections, the final questionnaire was written in Chinese and included demographic information, knowledge assessment, attitude evaluation, and practice analysis. The knowledge dimension employed the Coronary Artery Disease Education Questionnaire Short Version, which has been validated for assessing disease-related knowledge among cardiovascular patients and serves as an appropriate tool for evaluating cardiac rehabilitation content.^[15]^ Translation of the Coronary Artery Disease Education Questionnaire Short Version into Chinese was conducted by Yang Lamei and Luo Shilan, and its reliability was verified in a sample of 220 CHD patients, with an overall Cronbach α coefficient of 0.854. Each item in the scale is scored dichotomously, with 1 point awarded for a correct response and 0 points for incorrect or unknown answers, resulting in a maximum score of 20 points indicative of higher disease awareness. The attitude dimension comprised 5 questions rated on a 5-point Likert scale ranging from strongly agree (5 points) to strongly disagree (1 point), yielding a score range of 5 to 25 points. Similarly, the practice dimension consisted of 10 questions assessed on a scale from very positive behavior (5 points) to very negative behavior (1 point), with a score range of 10 to 50 points. Descriptive analysis was conducted for both sections 5.1 and 5.2. A scoring threshold of > 70% for each dimension was established to define adequate knowledge, positive attitudes, and proactive practices.^[16]^

### 2.4. Statistical analysis

Data analysis was conducted using SPSS 22.0 (IBM, Armonk). Continuous data are presented as means and standard deviations, while categorical data are expressed as n (%). Continuous variables underwent a normality test, with the *t*-test for normally distributed data and the Wilcoxon Mann–Whitney test for non-normally distributed data when comparing 2 groups. For 3 or more groups with normally distributed continuous variables and uniform variance, analysis of variance was used for comparisons, while the Kruskal–Wallis test was employed for non-normally distributed data. Structural equation modeling (SEM) was utilized to explore the relationships between knowledge (K), attitude (A), and practice (P). A two-sided *P*-value <.05 was considered statistically significant.

## 3. Results

Among the 490 participants, 292 (59.59%) were male, with mean age of 61.54 ± 11.01 years, 380 (77.55%) lived in rural areas, 355 (72.45%) had junior high school education or below, 355 (72.45%) had an average family monthly per capita income of <5000 Yuan, 301 (61.43%) suffered from one of the underlying diseases, 409 (83.47%) had angina pectoris, 157 (32.04%) had implanted a stent, and 157 (32.04%) were under regular treatment with CHD medication as prescribed by their doctors. The mean knowledge, attitude, and practice scores were 11.68 ± 5.21 (possible range: 0–20), 21.11 ± 2.62 (possible range: 5–25), and 39.97 ± 7.46 (possible range: 10–50), separately. The knowledge score varied from patients with different residence (*P* < .001), education (*P* < .001), and average family monthly per capita income (*P* < .001). As for the attitude score, there were difference among patients with different gender (*P* = .016), residence (*P* = .024), education (*P* = .021), average family monthly per capita income (*P* = .041), and the type of CHD (*P* = .005). The difference of practice score were found among patients with different marital status (*P* = .022), the type of CHD (*P* = .007), and whether they were implanted a stent (*P* = .004; Table 1).

**Table 1:** Demographic characteristics and KAP scores.

N = 490	N (%)	Knowledge score		Attitude score		Practice score	
		Mean ± SD	*P*	Mean ± SD	*P*	Mean ± SD	*P*
Total score		11.68 ± 5.21		21.11 ± 2.62		39.97 ± 7.46	
Gender			.120		.016		.467
Male	292 (59.59)	12 ± 5.06		21.30 ± 2.64		40.06 ± 7.78	
Female	198 (40.41)	11.21 ± 5.37		20.80 ± 2.56		39.83 ± 6.97	
Age (yrs)	61.54 ± 11.01						
Residence			<.001		.024		.850
a. Rural	380 (77.55)	11.12 ± 5.23		20.94 ± 2.69		39.90 ± 7.74	
b. Urban	110 (22.45)	13.60 ± 4.61		21.65 ± 2.28		40.20 ± 6.43	
Education			<.001		.021		.506
a. Junior high school or below	355 (72.45)	11.01 ± 5.25		20.91 ± 2.65		39.86 ± 7.74	
b. High school/technical school	73 (14.9)	12.54 ± 4.74		21.43 ± 2.42		39.89 ± 6.11	
c. College	37 (7.55)	14.02 ± 4.50		21.35 ± 2.53		39.83 ± 7.45	
d. Bachelor degree and above	25 (5.10)	15.16 ± 4.14		22.52 ± 2.31		41.96 ± 6.99	
Average family monthly per capita income, RMB			<.001		.041		.501
<5000	389 (79.39)	11.15 ± 5.25		20.96 ± 2.64		39.85 ± 7.58	
≥5000	101 (20.61)	13.70 ± 4.47		21.66 ± 2.46		40.44 ± 6.96	
Marital status			.141		.600		.022
a. Unmarried/Divorced/Widowed	24 (4.90)	9.833 ± 6.09		20.75 ± 3.33		36.62 ± 7.32	
b. Married	466 (95.1)	11.77 ± 5.14		21.12 ± 2.58		40.14 ± 7.43	
Number of underlying diseases			.467		.162		.465
0	8 (1.63)	13.87 ± 5.61		22.75 ± 2.05		41 ± 6.27	
1	301 (61.43)	11.87 ± 4.96		21.13 ± 2.51		40.37 ± 7.19	
2	153 (31.22)	11.32 ± 5.48		21.05 ± 2.89		39.45 ± 7.90	
≥3	28 (5.71)	11.03 ± 6.02		20.53 ± 2.09		38.14 ± 7.95	
Smoking			.716		.352		.479
Smoker	139 (28.37)	11.31 ± 5.33		21.26 ± 2.67		39.48 ± 8.19	
Never smoked	276 (56.33)	11.72 ± 5.35		20.99 ± 2.56		40.08 ± 6.78	
Quit smoking	75 (15.31)	12.22 ± 4.33		21.22 ± 2.72		40.46 ± 8.42	
Type of coronary heart disease			.584		.005		.007
a. Angina pectoris	409 (83.47)	11.64 ± 5.17		20.96 ± 2.61		39.59 ± 7.50	
b. Myocardial infarction	81 (16.53)	11.86 ± 5.37		21.81 ± 2.52		41.90 ± 6.97	
Coronary stent implantation?			.582		.094		.004
a. Yes, implanted stent	157 (32.04)	11.95 ± 4.82		21.37 ± 2.74		41.43 ± 6.77	
b. No stent implanted	333 (67.96)	11.55 ± 5.37		20.97 ± 2.55		39.28 ± 7.67	
Following doctor’s orders for regular coronary heart disease medication treatment?			.125		.080		.169
a. Yes	469 (95.71)	11.77 ± 5.15		21.15 ± 2.58		40.10 ± 7.31	
b. No	21 (4.29)	9.714 ± 5.95		19.90 ± 3.19		37 ± 9.90	

Types of medication you are taking (multiple selections allowed)							
a. Aspirin or clopidogrel	428 (87.35)						
b. Clopidogrel or ticagrelor	358 (73.06)						
c. Rivaroxaban	10 (2.04)						
d. Warfarin	1 (0.2)						
e. Isosorbide dinitrate	329 (67.14)						
f. Nitroglycerin	39 (7.96)						
g. Compound Danshen	57 (11.63)						
h. Statins such as atorvastatin	403 (82.24)						
i. Angiotensin receptor blockers or ACE inhibitors such as valsartan, lisinopril	207 (42.24)						
j. Metoprolol	162 (33.06)						
k. Antidiabetic drugs	146 (29.8)						
l. Diuretics such as torasemide, furosemide	17 (3.47)						

KAP = knowledge, attitudes, and practices, SD = standard deviation.

The distribution of knowledge dimension revealed that the question with the highest number of participants choosing the “a. correct” option were “Pickled foods (such as kimchi) are usually high in sodium.” (K9), with 85.31%. The question with the highest number of participants choosing the “b. incorrect” option were “If someone feels chest discomfort while walking, he or she should walk faster to see if the discomfort disappears.” (K13), with 66.33%. The question with the highest number of participants choosing the “c. not clear” option were “‘Statins’ limit the body’s intake of cholesterol from food. Statins include atorvastatin, rosuvastatin, or simvastatin.” (K11), with 45.51% (Table 2).

**Table 2:** Distribution of knowledge dimension responses.

Knowledge	N (%)		
	a. correct	b. incorrect	c. not clear
1. Coronary artery disease is a type of heart artery disease that occurs only in elderly people with high cholesterol or who smoke.	173 (35.31)	178 (36.33)	139 (28.37)
2. Modifiable cardiovascular disease risk factors include: blood pressure, high cholesterol, smoking and exposure to secondhand smoke, waist circumference, and response to stress, etc.	356 (72.65)	17 (3.47)	117 (23.88)
3. “Angina pectoris” is a chest pain or discomfort that occurs during rest or physical activity, and this pain can radiate to the arms, back, or neck.	359 (73.27)	17 (3.47)	114 (23.27)
4. The benefits of resistance training (weight lifting or using resistance bands) include: increasing strength, improving daily activity capacity, improving blood sugar levels, and increasing muscle mass.	303 (61.84)	26 (5.31)	161 (32.86)
5. Eating more meat and dairy products is a good way to increase dietary fiber.	205 (41.84)	139 (28.37)	146 (29.8)
6. Antiplatelet drugs like aspirin are important because they help prevent blood clots in the blood, reduce the “stickiness” of platelets in the blood, making it easier for blood to pass through the coronary arteries.	341 (69.59)	11 (2.24)	138 (28.16)
7. The only effective way to deal with stress is to avoid and stay away from people who cause unpleasant feelings.	282 (57.55)	120 (24.49)	88 (17.96)
8. Warm-up exercises gradually increase heart rate and can reduce the risk of angina.	245 (50)	72 (14.69)	173 (35.31)
9. Pickled foods (such as kimchi) are usually high in sodium.	418 (85.31)	7 (1.43)	65 (13.27)
10. Depression is common after a heart attack. Depression can lower a patient’s ability to recover later and increase the risk of another acute heart attack.	295 (60.2)	29 (5.92)	166 (33.88)
11. “Statins” limit the body’s intake of cholesterol from food. Statins include atorvastatin, rosuvastatin, or simvastatin.	253 (51.63)	14 (2.86)	223 (45.51)
12. To control blood pressure, people should limit daily salt intake to <6 grams, exercise regularly, take antihypertensive drugs as prescribed, and learn relaxation techniques.	373 (76.12)	6 (1.22)	111 (22.65)
13. If someone feels chest discomfort while walking, he or she should walk faster to see if the discomfort disappears.	66 (13.47)	325 (66.33)	99 (20.2)
14. Trans fats are harmful fats that are often found in fried or baked foods.	325 (66.33)	18 (3.67)	147 (30)
15. Untreated sleep apnea increases the risk of a recurrent heart attack but does not increase the risk of death.	98 (20)	197 (40.2)	195 (39.8)
16. To control high cholesterol, a person should become a vegetarian and avoid eating eggs.	112 (22.86)	256 (52.24)	122 (24.9)
17. When your heart rate is within the target range during exercise, and you feel that you are exerting effort but not “struggling,” and you can still talk while exercising, it means you are doing the right amount of exercise.	253 (51.63)	78 (15.92)	159 (32.45)
18. Exercise and a healthy diet cannot prevent diabetes.	87 (17.76)	288 (58.78)	115 (23.47)
19. Stress is an important risk factor for heart attacks, comparable to the risk of hypertension and diabetes.	333 (67.96)	27 (5.51)	130 (26.53)
20. A diet that helps lower blood pressure should be rich in: vegetables and fruits, whole grains, low-fat dairy products, nuts, etc.	368 (75.1)	15 (3.06)	107 (21.84)

In terms of attitudes, 46.94% were very willing to participate with their doctors in the development of their cardiac rehabilitation plan (A3), and 43.47% believed that cardiac rehabilitation was essential for subsequent recovery and quality of life and therefore needed to be approached with great care (A2). At the same time, 61.84% believe that cardiac rehabilitation has some risks and is not entirely safe (A1). In addition, 58.98% believe that cardiac rehabilitation may be needed for the rest of their lives (A5) (Table 3).

**Table 3:** Distribution of attitude dimension responses.

Attitude	Strongly agree	Agree	Neutral	Disagree	Strongly disagree
1. I think cardiac rehabilitation is not so safe and still has some risks.	112 (22.86)	303 (61.84)	29 (5.92)	40 (8.16)	6 (1.22)
2. I think cardiac rehabilitation is crucial for subsequent recovery and quality of life and needs to be treated with caution.	213 (43.47)	250 (51.02)	23 (4.69)	3 (0.61)	1 (0.2)
3. I am willing to participate with the doctor in formulating my cardiac rehabilitation plan, including cardiovascular function assessment, medication prescription, exercise prescription, nutrition prescription, psychological stress prescription, etc.	230 (46.94)	231 (47.14)	27 (5.51)	1 (0.2)	1 (0.2)
4. I am very concerned about the effectiveness or difficulty of cardiac rehabilitation for angina recovery and believe that more information and support are needed to make decisions, such as hospitals conducting more seminars on cardiac rehabilitation guidance.	173 (35.31)	278 (56.73)	35 (7.14)	4 (0.82)	/
5. I think cardiac rehabilitation may span my entire life.	138 (28.16)	289 (58.98)	36 (7.35)	27 (5.51)	/

Responses to the practice dimension showed that 62.45% always abstained from smoking and limiting alcohol (P4), and 55.71% always took their CHD medication as prescribed by their doctor (P2). On the other hand, 34.9% exercised daily (P5.1) and 48.16% exercised for half an hour every time (P5.2). It is noteworthy that 34.29% of the participants were only sometimes able to initiate their own cardiac rehabilitation plan (P1) (Table 4).

**Table 4:** Distribution of practice dimension responses.

Practice	Always	Often	Sometimes	Rarely	Never
1. I can actively implement my own cardiac rehabilitation plan.	95 (19.39)	158 (32.24)	168 (34.29)	60 (12.24)	9 (1.84)
2. I can regularly take coronary artery disease medications as prescribed in daily life.	273 (55.71)	135 (27.55)	56 (11.43)	20 (4.08)	6 (1.22)
3. I can control my daily salt intake in daily life.	138 (28.16)	176 (35.92)	118 (24.08)	51 (10.41)	7 (1.43)
4. I can quit smoking and limit alcohol intake in daily life.	306 (62.45)	85 (17.35)	61 (12.45)	29 (5.92)	9 (1.84)
5. I will engage in moderate physical exercise according to the doctor’s advice to improve cardiovascular function and physical fitness.	195 (39.8)	159 (32.45)	100 (20.41)	27 (5.51)	9 (1.84)
	a. Exercise every day	b. 4–5 times a wk	c. 1–2 times a wk	d. Do not exercise	
5.1 Number of exercise sessions per week	171 (34.9)	122 (24.9)	136 (27.76)	61 (12.45)	
	a. <30 min	b. 30 min–1 h	c. >1 h		
5.2 Duration of each exercise session	236 (48.16)	214 (43.67)	40 (8.16)		
6. I can maintain a balanced diet and healthy eating habits in daily life.	161 (32.86)	172 (35.1)	119 (24.29)	32 (6.53)	6 (1.22)
7. I can maintain regular daily routines and avoid staying up late.	184 (37.55)	162 (33.06)	105 (21.43)	26 (5.31)	13 (2.65)
8. I can maintain a positive and optimistic attitude in daily life (during cardiac rehabilitation).	190 (38.78)	175 (35.71)	100 (20.41)	19 (3.88)	6 (1.22)
9. I will try to avoid factors that trigger angina attacks, such as vigorous exercise, extreme temperatures, and emotional fluctuations.	171 (34.9)	182 (37.14)	100 (20.41)	29 (5.92)	8 (1.63)
10. I will undergo regular checkups and adjust the cardiac rehabilitation plan with the doctor.	201 (41.02)	150 (30.61)	90 (18.37)	42 (8.57)	7 (1.43)

Correlation analyses indicated significant positive correlations between knowledge and attitude (*R* = 0.3210, *P* < .001), as well as practice (*R* = 0.3019, *P* < .001). Meanwhile, there was also correlation between attitude and practice (*R* = 0.3521, *P* < .001) (Table 5).

**Table 5:** Correlation analysis.

	Knowledge	Attitude	Practice
Knowledge	1		
Attitude	0.3210 (*P* < .001)	1	
Practice	0.3019 (*P* < .001)	0.3521 (*P* < .001)	1

The distribution of scores showed that 405 (82.65%), 240 (48.98%), and 236 (48.16%) scored no >80% of the maximum score for knowledge, attitude, and practice, respectively (Table 6). Multivariate logistic regression showed that college education (OR = 2.76, 95% CI: [1.17–6.54], *P* = .02) was independently associated with good knowledge (Table 7). Meanwhile, knowledge score (OR = 1.11, 95% CI: [1.07–1.15], *P* < .001), being female (OR = 0.62, 95% CI: [0.42–0.92], *P* = .017), and suffering from myocardial infarction (OR = 1.78, 95% CI: [1.05–3.00], *P* = .031) were independently associated with attitude (Table 8). Furthermore, knowledge score (OR = 1.08, 95% CI: [1.04–1.13], *P* < .001), attitude score (OR = 1.17, 95% CI: [1.08–1.27], *P* < .001), married (OR = 5.24, 95% CI: [1.76–15.6], *P* = .003), and suffering from myocardial infarction (OR = 1.86, 95% CI: [1.09–3.19], *P* = .023) were independently associated with proactive practice (Table 9).

**Table 6:** Score distribution.

	Knowledge	Attitude	Practice
≤80%	405 (82.65)	240 (48.98)	236 (48.16)
>80%	85 (17.35)	250 (51.02)	254 (51.84)

**Table 7:** Univariate and multivariate analysis for knowledge dimension.

Knowledge	Univariate analysis			Multivariate analysis	
	OR (95% CI)	*P*	OR (95% CI)		*P*
Gender					
Male					
Female	0.72 (0.44–1.18)	.195			
Age (yrs)	0.99 (0.97–1.01)	.591			
Residence					
Rural					
Urban	2.53 (1.52–4.18)	<.001	1.30 (0.64–2.63)		.463
Education					
Junior high school or below					
High school/ technical school	1.88 (0.99–3.55)	.05	1.31 (0.60–2.84)		.49
College	4.08 (1.96–8.51)	<.001	2.76 (1.17–6.54)		.02
Bachelor degree and above	3.77 (1.57–9.04)	.003	2.33 (0.82–6.55)		.108
Average family monthly per capita income, RMB					
<5000					
≥5000	2.74 (1.64–4.58)	<.001	1.57 (0.80–3.10)		.186
Marital status					
a. Unmarried/Divorced/Widowed					
b. Married	1.49 (0.43–5.12)	.523			
Number of underlying diseases					
0					
1	0.37 (0.08–1.60)	.185			
2	0.29 (0.06–1.31)	.11			
≥3	0.27 (0.04–1.64)	.158			
Smoking					
Smoker					
Never smoked	1.44 (0.82–2.53)	.195			
Quit smoking	1.02 (0.46–2.26)	.956			
Type of coronary heart disease					
a. Angina pectoris					
b. Myocardial infarction	0.99 (0.52–1.86)	.987			
Coronary stent implantation?					
a. Yes, implanted stent					
b. No stent implanted	1.33 (0.79–2.24)	.28			
Following doctor’s orders for regular coronary heart disease medication treatment?					
a. Yes					
b. No	0.78 (0.22–2.73)	.706			

**Table 8:** Univariate and multivariate analysis for attitude dimension.

Attitude	Univariate analysis			Multivariate analysis	
	OR (95% CI)	*P*	OR (95% CI)		*P*
Knowledge score	1.11 (1.07–1.16)	<.001	1.11 (1.07–1.15)		<.001
Gender					
Male					
Female	0.57 (0.40–0.83)	.003	0.62 (0.42–0.92)		.017
Age (yrs)	0.99 (0.97–1.00)	.286			
Residence					
Rural					
Urban	1.45 (0.94–2.22)	.089			
Education					
Junior high school or below					
High school/ technical school	1.24 (0.75–2.06)	.389	1.03 (0.60–1.75)		.9
College	2.26 (1.10–4.65)	.026	1.42 (0.66–3.06)		.36
Bachelor degree and above	1.93 (0.83–4.49)	.125	1.12 (0.46–2.74)		.787
Average family monthly per capita income, RMB					
<5000					
≥5000	1.31 (0.84–2.04)	.223			
Marital status					
a. Unmarried/Divorced/Widowed					
b. Married	1.24 (0.54–2.83)	.603			
Number of underlying diseases					
0					
1	0.35 (0.07–1.78)	.208			
2	0.35 (0.06–1.81)	.214			
≥3	0.18 (0.03–1.09)	.063			
Smoking					
Smoker					
Never smoked	0.69 (0.46–1.04)	.084			
Quit smoking	0.99 (0.56–1.75)	.987			
Type of coronary heart disease					
a. Angina pectoris					
b. Myocardial infarction	1.79 (1.09–2.93)	.02	1.78 (1.05–3.00)		.031
Coronary stent implantation?					
a. Yes, implanted stent					
b. No stent implanted	0.74 (0.50–1.08)	.127			
Following doctor’s orders for regular coronary heart disease medication treatment?					
a. Yes					
b. No	0.46 (0.18–1.17)	.105			

**Table 9:** Univariate and multivariate analysis for practice dimension.

Practice	Univariate analysis		Multivariate analysis	
	OR (95% CI)	*P*	OR (95% CI)	*P*
Knowledge score	1.11 (1.07–1.15)	<.001	1.08 (1.04–1.13)	<.001
Attitude score	1.23 (1.14–1.32)	<.001	1.17 (1.08–1.27)	<.001
Gender				
Male				
Female	0.97 (0.68–1.40)	.907		
Age (yrs)	0.99 (0.98–1.01)	.842		
Residence				
Rural				
Urban	0.90 (0.59–1.39)	.662		
Education				
Junior high school or below				
High school/technical school	0.73 (0.44–1.21)	.228		
College	0.67 (0.34–1.33)	.263		
Bachelor degree and above	1.88 (0.79–4.48)	.15		
Average family monthly per capita income, RMB				
<5000				
≥5000	1.14 (0.73–1.77)	.555		
Marital status				
a. Unmarried/Divorced/Widowed				
b. Married	4.36 (1.60–11.8)	.004	5.24 (1.76–15.6)	.003
Number of underlying diseases				
0				
1	1.86 (0.43–7.94)	.399		
2	1.77 (0.41–7.70)	.441		
≥3	1.44 (0.28–7.24)	.655		
Smoking				
Smoker				
Never smoked	1.17 (0.77–1.76)	.445		
Quit smoking	1.44 (0.82–2.54)	.203		
Type of coronary heart disease:				
a. Angina pectoris				
b. Myocardial infarction	1.83 (1.12–3.01)	.016	1.86 (1.09–3.19)	.023
Coronary stent implantation?				
a. Yes, implanted stent				
b. No stent implanted	0.69 (0.47–1.01)	.063		
Following doctor’s orders for regular coronary heart disease medication treatment?				
a. Yes				
b. No	0.55 (0.22–1.37)	.203		

**Figure 1.**
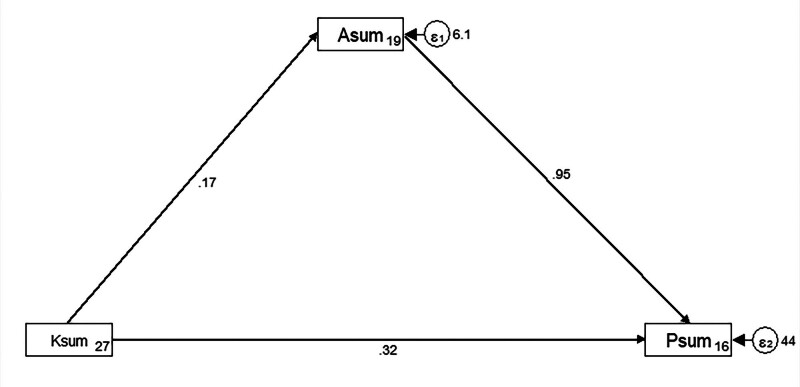
SEM model. SEM = structural equation modeling.

The SEM demonstrate a highly favorable model fit indices, suggesting a well-fitting model (Table 10), and shown that knowledge had direct effects on attitude (β = 0.16, *P* < .001) and practice (β = 0.32, *P* < .001). Moreover, attitude have a direct impact on practice (β = 0.95, *P* < .001). Furthermore, knowledge also had an indirect effect on practice through attitude (β = 0.15, *P* < .001) (Table 11 and Fig. 1).

**Table 10:** SEM model fit.

Indicators	Reference	Results
RMSEA	<0.08 Good	0.000
SRMR	<0.08 Good	0.000
TLI	>0.8 Good	1.000
CFI	>0.8 Good	1.000

CFI = Comparative Fit Index, RMSEA = root mean square error of approximation, SEM = structural equation modelling, SRMR = standardized root mean square residual, TLI = Tucker–Lewis Index.

**Table 11:** Results of SEM analysis.

Model paths	Total effects		Direct effect		Indirect effect	
	β (95% CI)	*P*	β (95% CI)	*P*	β (95% CI)	*P*
Asum <-						
Ksum	0.16 (0.12–0.20)	<.001	0.16 (0.12–0.20)	<.001		
Psum <-						
Asum	0.95 (0.71–1.19)	<.001	0.95 (0.71–.19)	<.001		
Ksum	0.48 (0.36–0.60)	<.001	0.32 (0.20–0.44)	<.001	0.15 (0.10–0.21)	<.001

SEM = structural equation modeling.

## 4. Discussion

The study reveals that CHD patients exhibit insufficient knowledge, yet demonstrate positive attitudes and proactive practices towards cardiac rehabilitation. Given the findings, it is recommended that healthcare providers prioritize educational interventions aimed at enhancing CHD patients’ knowledge about cardiac rehabilitation, while also leveraging and reinforcing their existing positive attitudes and proactive practices to optimize their engagement in rehabilitation programs.

The findings of the study reveal a nuanced perspective on the KAP of individuals with CHD towards cardiac rehabilitation. Given the endorsement of cardiac rehabilitation benefits by multiple guidelines, many healthcare professionals may recommend this lifestyle strategy to patients, leading to patients exhibiting positive attitudes and practices despite lacking comprehensive knowledge.^[17,18]^

In this study, gender emerged as a significant factor affecting attitudes towards cardiac rehabilitation in both inter-group comparisons and logistic regression analysis. Males displayed slightly higher attitude scores compared to females. This finding aligns with previous research suggesting that gender differences influence health-seeking behaviors and attitudes towards healthcare utilization.^[19,20]^ Possible explanations for this gender disparity may include differences in healthcare-seeking behavior, social roles, and cultural norms surrounding illness perception and treatment adherence. Addressing gender-specific barriers through targeted interventions and educational programs may help mitigate disparities in attitudes towards cardiac rehabilitation.

Furthermore, education level consistently emerged as a significant predictor of knowledge and attitudes towards cardiac rehabilitation. Individuals with a bachelor degree or higher exhibited higher knowledge and attitude scores in both inter-group comparisons and logistic regression analysis. This finding is consistent with the literature highlighting the role of education in health literacy and self-management behaviors.^[21]^ Higher educational attainment is associated with better access to health information, critical thinking skills, and understanding of health-related concepts, which may contribute to more positive attitudes towards rehabilitation. Efforts to improve health literacy through targeted educational interventions and accessible health information resources are essential for enhancing engagement with cardiac rehabilitation programs among individuals with lower educational levels.

The association between college education and good knowledge underscores the importance of formal education in promoting health literacy. College-educated individuals were more likely to possess adequate knowledge about cardiac rehabilitation, highlighting the role of educational attainment as a determinant of health-related knowledge. However, it’s crucial to recognize that education alone may not fully address disparities in health literacy, as other socioeconomic factors such as income and access to healthcare services also play significant roles.^[22]^ Therefore, interventions targeting health literacy should consider broader social determinants of health to effectively address disparities in knowledge and understanding of cardiac rehabilitation.

In correlation analyses and SEM, positive correlations were observed between KAP towards cardiac rehabilitation. These findings suggest that individuals with higher knowledge about rehabilitation tend to exhibit more positive attitudes and engage in proactive practices. This aligns with the theoretical framework of the health belief model, which posits that individuals are more likely to engage in health-promoting behaviors if they perceive themselves to be at risk and believe in the effectiveness of preventive actions.^[23,24]^ By elucidating the interrelationships between cognitive, affective, and behavioral components, these findings underscore the importance of addressing multiple dimensions of patient perceptions in promoting adherence to cardiac rehabilitation programs.

The analysis of the knowledge dimension reveals a mixed understanding among participants regarding various aspects of CHD management. While some concepts are well-understood by participants, such as modifiable cardiovascular risk factors and the benefits of certain medications, there are notable gaps in knowledge concerning dietary recommendations and symptom management. To address these gaps, healthcare providers should prioritize tailored educational interventions that focus on practical strategies for implementing dietary modifications and recognizing symptoms of CHD.^[25,26]^ Utilizing interactive educational formats, such as workshops or peer support groups, may enhance comprehension and retention of key concepts. Additionally, incorporating culturally relevant examples and real-life scenarios can help contextualize information and empower individuals to make informed decisions about their health.

The attitudes towards cardiac rehabilitation among participants reflect a spectrum of perceptions. Participants exhibited a range of attitudes towards cardiac rehabilitation, with some expressing strong support for its importance in recovery and quality of life, while others harbored concerns about its safety and effectiveness. To foster a more positive attitude towards cardiac rehabilitation, healthcare providers should prioritize patient education and communication to address concerns and misconceptions. Engaging patients in shared decision-making and providing comprehensive information about the benefits and safety of rehabilitation programs can help build trust and confidence in the rehabilitation process. Moreover, offering opportunities for peer support and mentorship can provide emotional reassurance and practical guidance, facilitating a more supportive environment for individuals undergoing cardiac rehabilitation.^[27,28]^

Adherence to recommended self-care behaviors varied among participants, with some demonstrating consistent engagement, particularly in medication adherence and regular checkups, while others showed opportunities for improvement in implementing personalized rehabilitation plans and maintaining lifestyle modifications. To promote consistent adherence to self-care behaviors, healthcare providers should offer personalized counseling and support to address individual barriers and facilitate behavior change. Implementing structured behavior change programs, such as motivational interviewing or cognitive-behavioral therapy, can empower individuals to overcome challenges and develop sustainable self-care practices. Moreover, leveraging technology-based platforms, such as mobile applications or telemedicine, can enhance accessibility and support ongoing monitoring and adherence to recommended behaviors. By addressing these challenges and providing targeted support, healthcare providers can optimize patient engagement with cardiac rehabilitation and improve outcomes for individuals with CHD.^[29,30]^

Despite the valuable insights gained, this study has several limitations. Firstly, its cross-sectional design limits the ability to establish causality between variables, providing only a snapshot of the relationships observed at a single point in time. Secondly, the reliance on self-reported data through questionnaires may introduce response bias and inaccuracies due to recall or social desirability biases. Thirdly, the study was conducted in a single hospital setting, which may limit the generalizability of the findings to other populations or settings. These limitations warrant cautious interpretation of the results and emphasize the need for further research employing longitudinal designs and diverse participant samples to validate and expand upon the current findings. Additionally, the study did not distinguish between patients at different stages of the cardiac rehabilitation process (e.g., rehabilitation-naïve vs in-progress), nor did it collect detailed information regarding revascularization status following coronary angiography. These factors may influence patients’ KAP, and should be considered in future studies.

In conclusion, CHD patients in this study demonstrated inadequate knowledge, yet exhibited positive attitudes and proactive practices towards cardiac rehabilitation. It is imperative for healthcare providers to implement targeted educational interventions aimed at improving knowledge levels among CHD patients, which may further enhance their attitudes and practices towards cardiac rehabilitation, ultimately contributing to improved clinical outcomes. Future studies should adopt longitudinal designs to observe the evolution of KAP across different stages of cardiac rehabilitation and incorporate clinical data such as revascularization status. Interventions targeting specific knowledge gaps, especially among patients with low educational attainment, should be developed and evaluated for their impact on behavioral outcomes and adherence.

## Author contributions

**Conceptualization:** Weifeng Zhang, Zesheng Xu, Xinwei Jia.

**Data curation:** Weifeng Zhang, Zesheng Xu, Yeran Zhu, Xinwei Jia.

**Formal analysis:** Weifeng Zhang, Zesheng Xu, Jianjun Chen, Xinwei Jia, Yanmin Wu.

**Funding acquisition:** Zesheng Xu.

**Investigation:** Weifeng Zhang, Jing Zhang, Yugang Zu.

**Methodology:** Weifeng Zhang, Zesheng Xu, Yanling Li, Haiyan Jia.

**Project administration:** Yanling Li, Haiyan Jia, Jing Zhang, Yugang Zu.

**Software:** Yanling Li.

**Supervision:** Yugang Zu.

**Validation:** Zesheng Xu.

**Writing – original draft:** Weifeng Zhang.

**Writing – review & editing:** Weifeng Zhang, Jianjun Chen.
